# Autumn migration direction of juvenile willow warblers (*Phylloscopus t. trochilus* and *P. t. acredula*) and their hybrids assessed by qPCR SNP genotyping

**DOI:** 10.1186/s40462-020-00209-7

**Published:** 2020-05-29

**Authors:** Tianhao Zhao, Mihaela Ilieva, Keith Larson, Max Lundberg, Júlio M. Neto, Kristaps Sokolovskis, Susanne Åkesson, Staffan Bensch

**Affiliations:** 1 Department of Biology, Molecular Ecology and Evolution Laboratory, Lund University, Ecology Building, 223 62 Lund, SE Sweden; 2 Institute of Biodiversity and Ecosystem Research, Bulgarian Academy of Sciences, 2 Gagarin str, 1113 Sofia, Bulgaria; 3 Climate Impacts Research Centre, Department of Ecology and Environmental Sciences, Umeå University, 901 87 Umeå, SE Sweden; 4 Department of Biology, Evolutionary Ecology Unit, Lund University, Ecology Building, 223 62 Lund, SE Sweden

**Keywords:** Bird migration, Genetic migration program, Migratory divide, Intermediate route, SNP genotyping, Hybrid genotype, *Phylloscopus trochilus*

## Abstract

**Backgrounds:**

Geographic regions, where two closely related taxa with different migration routes come into contact, are known as migratory divides. Hybrids originating from migratory divides are hypothesized to migrate intermediately relative to the parental populations. Few studies have tested this hypothesis in wild birds, and only in hybrids that have completed the migration back to the breeding grounds. Here, we make use of the well-established migration routes of willow warblers (*Phylloscopus trochilus*), for which the subspecies *trochilus* and *acredula* have migration-associated genetic markers on chromosomes 1 and 5. The genetic approach enabled us to analyze the geographic distribution of juveniles during their first autumn migration, predicting that hybrids should be more frequent in the central flyway over Italy than along the typical SW routes of *trochilus* and SE routes of *acredula*.

**Methods:**

Blood and feather samples were collected from wintering birds in Africa (*n* = 69), and from juveniles during autumn migration in Portugal (*n* = 33), Italy (*n* = 38) and Bulgaria (*n* = 32). Genotyping was carried out by qPCR SNP assays, on one SNP each on chromosome 1 (SNP 65) and chromosome 5 (SNP 285). Both these SNPs have alternative alleles that are highly fixed (> 97%) in each of the subspecies.

**Results:**

The observed combined genotypes of the two SNPs were associated with the known migration routes and wintering distributions of *trochilus* and *acredula*, respectively. We found hybrids (HH) among the juveniles in Italy (5/38) and in Portugal (2/33). The proportion of hybrids in Italy was significantly higher than expected from a background rate of hybrid genotypes (1.5%) in allopatric populations of the subspecies.

**Conclusions:**

Our genetic approach to assign individuals to subspecies and hybrids allowed us to investigate migration direction in juvenile birds on their first migration, which should better reflect the innate migratory direction than studies restricted to successful migrants. The excess of hybrids in Italy, suggests that they employ an intermediate route relative to the parental populations. Our qPCR SNP genotyping method is efficient for processing large sample sizes, and will therefore be useful in migration research of species with known population genetic structure.

## Introduction

Migratory divides, defined as geographic regions where the ranges of two closely related taxa that differ in migration routes come into contact and interbreed, are described from several avian species in Europe [1], Asia [2] and North America [3]. It has been proposed that migratory divides are maintained by selection against hybrids [1, 3–5]. This idea is based on the hypothesis that hybrids migrate along an intermediate direction relative to their parents, and that such routes confer lower survival, requiring hybrids to cross major geographical barriers, e.g. oceans (Mediterranean Sea [6]), deserts (Sahara [7]) or mountain ranges (Qinghai-Tibetan Plateau [2]).

The “intermediate route” hypothesis has been investigated by various methods. The classic studies of experimentally cross-bred blackcaps from populations with SW and SE migration, respectively, used orientation cages to demonstrate that F1 hybrids exhibited an intermediate direction towards south [8]. However, initial migration directions measured in orientation experiments may not be representative to the migration routes of hybrids in the wild, and thus other methods are required for obtaining data on migration routes and wintering areas. The first attempts to investigate the migration routes of hybrids in the wild made use of stable isotope analyses of feathers moulted at the wintering grounds [5, 9, 10]. These studies suggested that hybrids between two species of flycatchers and between *Acrocephalus* warblers occupied wintering areas, and presumably used migration routes, that were similar to one of the parental species rather than migrating intermediately. However, these results should be cautiously interpreted because stable isotype analyses are known to have low precision when predicting moulting areas of individual birds [5, 10, 11] and the link between migration routes and wintering areas may not be straightforward. More detailed information about the migration routes was obtained by Delmore et al. (2014) who used light-level geolocators to track 15 hybrids between the coastal and inland subspecies of the Swainson’s thrush (*Catharus ustulatus*), two populations previously documented to show different migration routes and wintering areas [12, 13]. About half of the hybrids displayed an intermediate migratory behavior, providing the strongest evidence so far for the “intermediate route” hypothesis. However, studies based on stable isotope analyses and tracks from geolocators have the inherited constraint that the wintering areas and migration routes can only be obtained from successfully migrating individuals, i.e. those that managed to return from the winter quarters to the breeding sites. If selection operates against first-time (juvenile) migrants employing the intermediate route, these will be underestimated in any study based on analyses of returning birds. Among other methodologies for studying migration, GPS transmitters are hitherto too big for most of the songbirds [14]. Telemetry studies require a dense network of e.g. MOTUS receivers to function in a large geographic range to provide meaningful route details [15], which is not yet in place for studies of songbirds that have migratory divides. Hence, other approaches are needed to obtain unbiased estimates of migration routes taken by first-time juvenile migratory birds, e.g. genetic tools [16, 17].

**Fig. 1:**
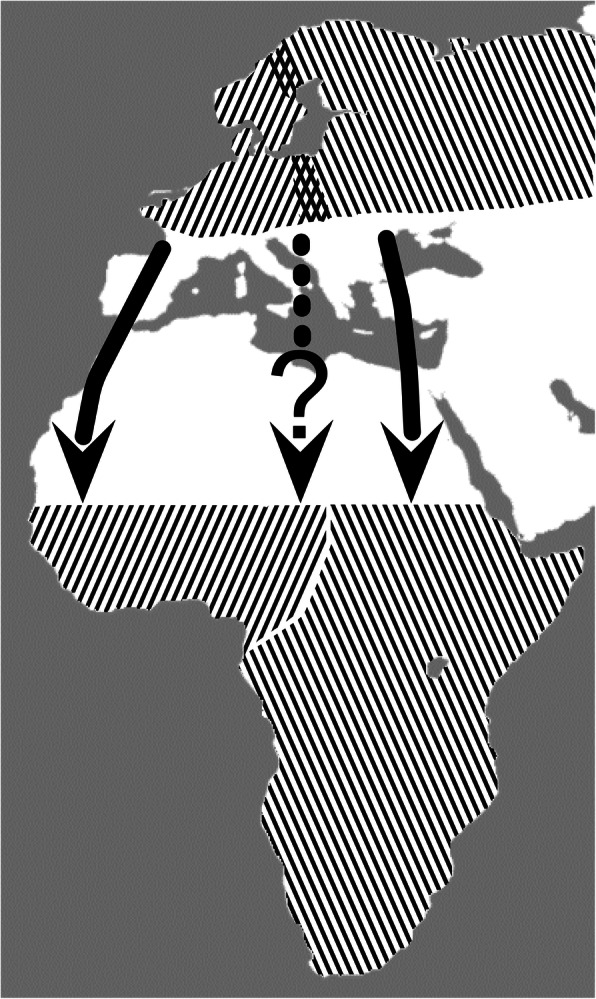
Approximate breeding and wintering ranges of the two subspecies of willow warblers, and their contact zones (migratory divides) in Scandinavia and Poland indicated by the range overlap. The subspecies *trochilus* migrates SW through Iberian Peninsula and winters in western Africa, whereas subspecies *acredula* migrates SSE through eastern Europe and winters in eastern to southern Africa. The hybrids originating from the migratory divides are hypothesized to migrate intermediately relative to the parental routes

The willow warbler *Phylloscopus trochilus* is a small-sized long-distance migratory songbird that breeds in the northern parts of the Palearctic from western Europe to eastern Siberia [18]. Its wintering grounds are located in sub-Saharan Africa as supported by ringing recoveries [19] and tracks obtained from light-level geolocators [20, 21]. Two subspecies occur in the western part of its range; *P. t. trochilus* and *P. t. acredula*. Despite slight but significant average differences in coloration and morphometrics, individuals cannot accurately be assigned to subspecies (or hybrids) due to extensive overlap in the phenotype [22]. The subspecies *trochilus* breeds in western Europe and southern Scandinavia, and migrates through western Europe to wintering grounds in western Africa. The subspecies *acredula* breeds in eastern Europe and northern Scandinavia and migrates through eastern Europe to wintering grounds in eastern and southern Africa. The *trochilus* and *acredula* populations meet in a well-defined contact zone in central Scandinavia and in a broader more diffuse zone in Poland [22] (Fig. 1).

The willow warbler has been a focal species for research on genetics of migration for two decades [21–28]. Stable isotope analyses (δ^15^N) of winter moulted feathers have revealed that there is a distinct migratory divide in central Scandinavia [22, 23, 28] where the two subspecies mate at random [27] and hybrids are frequent [28]. In the migratory divide, the population average δ^15^N is between the values of allopatric *trochilus* and *acredula*, respectively [23]. However, this data cannot distinguish whether hybrids are migrating intermediately relative the parental forms or whether the migratory divide consist of a mix of birds with either typical *trochilus* or *acredula* migratory directions.

The genomes between the subspecies are virtually undifferentiated except in three well-defined chromosome regions [25, 28]. The very limited recombination between the subspecies in these regions gives rise to sets of long-range northern and southern haplotypes. The geographic distributions of the northern and southern haplotypes in two of the divergent regions (chromosome 1 and 5) perfectly match the location of the migratory divides, suggesting that these genomic regions contain genes associated with the migratory behavior [28]. The highly fixed SNPs (Single Nucleotide Polymorphisms) in each of these regions can therefore be used to assign individuals to each subspecies (*trochilus* and *acredula*) and to identify hybrids.

In this study, we investigated how juvenile willow warblers migrate in their first autumn migration by using SNP genotyping methods based on qPCR (quantitative Polymerase Chain Reaction). This methodology allows us to identify subspecies and hybrids among birds caught during the non-breeding season. We first tested the power of the assay by analyzing samples of willow warblers from the African wintering grounds with the expectation to find the *trochilus* genotypes in western Africa and the *acredula* genotype in eastern and southern Africa. Next, we set up three predictions for the juvenile birds captured during autumn migration. (1) Samples from western Europe (Portugal) will be dominated by the “*trochilus*” genotype. (2) Samples from eastern Europe (Bulgaria) will be dominated by the *acredula* genotype. (3) If F1 hybrid juveniles between *acredula* and *trochilus* exist, they will be over-represented in central southern Europe (Italy) according to the intermediate route hypothesis. This approach circumvents the limitations of stable isotope and geolocator studies, as willow warblers were sampled during their first autumn migration before reaching the major ecological barriers (the Mediterranean Sea and the Sahara Desert) that presumably cause greater mortality to the maladapted hybrids and thereby will provide an accurate measure of realized migratory directions of first-year hybrids.

## Methods and materials

### Study species and sampling locations

We genotyped individual willow warblers from blood and feather DNA samples collected from breeding grounds in Scandinavia, during autumn migration at three locations in southern Europe and on wintering grounds in Africa (Table S1). To determine the genotype frequencies of allopatric *trochilus* and *acredula*, we used samples from previous publications [22] that consisted of territorial males from southern Scandinavia (Denmark and southern Sweden, latitude between 55°-60°, *n* = 66) and northern Scandinavia (Finland, Norway and northern Sweden, latitude above 65°, *n* = 60); both data sets are from areas well outside the contact zones. The autumn migration samples were collected between August–October in Portugal (*n* = 33), Italy (*n* = 38) and Bulgaria (*n* = 32). The samples from the wintering range (November–April) were collected in Ivory Coast (*n* = 12), Cameron (*n* = 27), Kenya (*n* = 3), Tanzania (*n* = 4), Zambia (*n* = 15) and South Africa (*n* = 8).

### DNA extraction and quantification

Birds were captured in mist-nets and from each individual ~ 20–50 μl of blood was taken from the brachial vein and stored in SET buffer (0.15 M NaCl, 0.001 M EDTA and 0.05 M Tris). Genomic DNA was extracted using an ammonium acetate protocol [29] and diluted to a concentration of ~ 1 ng/μl.

The base of feather samples (~ 5 mm of either tail feathers or innermost primary flight feathers) were cut into two strips to expose the inner structure, and added to tubes with 100 μl lysis buffer and 1.5 proteinase K and incubated at 56 °C for 3 h with regular shaking every hour. Following the digestion, 10 μl Sodium Acetate (NaAc) (3 M) and 220 μl 95% cold ethanol was used to precipitate the DNA. The DNA was pelleted by high speed centrifugation and the pellet was washed in 100 μl 70% cold ethanol. The extracted DNA was dissolved in 1xTE and diluted to a concentration of ~ 2 ng/μl.

### SNP selection

A previous study based on whole-genome resequencing and genotyping by a SNP array [28] identified two large blocks on chromosome 1 (13 Mb) and chromosome 5 (4 Mb) that carried highly divergent and non-recombining haplotypes between southern (*trochilus*) and northern populations (*acredula*). From the set of variants called from the resequencing data of nine southern and nine northern willow warblers [28], we first selected four highly differentiated SNPs (two per chromosome) for developing qPCR assays for differentiating *trochilus* and *acredula*. We named the two on chromosome 1 “23” and “65”, and the two on chromosome 5 “285” and “412”, corresponding to the scaffolds on which they were located (Figure S1). All the selected SNPs were located within regions of 200 bp that in the resequencing data contained very few SNPs, in order to avoid having primers and probes overlapping polymorphic sites.

### Primer and probes

We used Thermo Fisher’s online Custom TaqMan® Assay Design Tool to design the probes and primers for all the four SNPs. For each SNP, a 400 bp sequence with the target SNP in the middle was submitted to the server, in which all other irrelevant SNPs were marked as “N”. The tool then automatically generated the forward and reverse primers as well as the probes to detect different alleles of the target SNPs. Fluorophores attached to the beacons to differentiate alleles were FAM and VIC. The designed primers and probes, are given in Table S2.

### SNP genotyping

The genotyping of the target SNPs where done using either of two different qPCR instruments; a MX3005P (Stratagene, La Jolla, CA, USA) or a BioRad CFX96™ Real-time PCR system (Bio-Rad Laboratories, CA, USA). On both instruments we used the universal Fast-two-steps protocol (95 °C, 15 min – 40*(95 °C, 10 s - 60 °C, 30 s, plate read)).

Samples were run in 10 μl volumes with 5 μl TaqMan® Genotyping Master Mix, 0.5 μl 20X genotype assay (containing primers and probes) and 4.5 μl diluted DNA samples (~ 2 ng/μl for feather DNA samples and ~ 1 ng/μl for blood DNA samples). We first tested the genotype assays on DNA from both blood and feathers for a limited number of samples (*n* = 8) with duplicates, to examine the consistency and success rate of this strategy. We also examined blood and feather DNA samples from the same individuals (*n* = 21) to confirm the repeatability of DNA sample from different resources. The qPCRs for all the samples in the present study were run on 96-plates including both negative and two positive controls. The positive controls consisted of samples with genotypes fixed for either of the two different alleles.

We used the allelic discrimination functions provided by the software of the two qPCR instruments to generate the scatterplots of the fluorescence signals; Bio-Rad CFX Maestro™ 1.1 software and Mx3005P qPCR software, respectively. Examples of the results are illustrated in Figure S2a (Bio-Rad CFX) and Figure S2b (Mx3005P).

### Consistency and fixation levels of the analyzed SNPs

We used the above mentioned pure *trochilus* and *acredula* breeding samples from Scandinavia to evaluate how well the single SNPs can be used to predict the haplotype assignment on the regions of chromosomes 1 and 5, as well as their fixation level, i.e. allele frequencies in the two populations. From the SNP array [28], each individual had previously been classified as northern homozygous, southern homozygous or heterozygous for the regions on chromosome 1 and 5 based on a multidimensional scaling (MDS) analysis of 108 and 31 SNPs, respectively. We found that the single SNPs genotyped by qPCR were generally good at predicting the haplotypes assigned by the SNP array [28] (Table S3). The consistency ratios for chromosome 1 were 95.9% for SNP 23 and 99.0% for SNP 65. For chromosome 5, both SNP 285 and SNP 412 had a consistency ratio of 97.3%. This demonstrates that each of the selected SNPs has a high power to correctly assign the haplotype blocks as *trochilus* and *acredula*, respectively.

All the four investigated SNPs showed a high level of fixation of the presumed subspecies-specific alleles (Table 1, Figure S3). Based on these results (consistency rates with the SNP array and fixation levels), we selected SNP 65 on chromosome 1 and SNP 285 on chromosome 5 for population assignment. The other two SNPs were used for verifying hybrid genotypes.

**Table 1:** Allele frequencies of the four selected SNPs in southern and northern Scandinavian populations of willow warblers

SNP	Allele frequencies	
	Southern population (*n* = 66)	Northern population (*n* = 60)
23 (T / G)	0.955 / 0.045	0.050 / 0.950
65 (C / A)	0.985 / 0.015	0.050 / 0.950
285 (A / G)	0.970 / 0.030	0.084 / 0.916
412 (G / A)	0.963 / 0.037	0.091 / 0.908

SNP 23 and 65 are located on chromosome 1, SNP 285 and 412 on chromosome 5, respectively

### Assignment of genotypes

Our selected SNPs were all bi-allelic, so for each SNP locus there are three possible genotypes, denoted by N for the northern homozygous genotype, H for the heterozygous genotype, and S for the southern homozygous genotype. As we were using two SNPs (65 from chromosome 1 and 285 from chromosome 5), there are 9 possible combined genotypes: NN, NH, HN, NS, HH, SN, SH, HS and SS. We used the allele frequencies in the southern and northern Scandinavian willow warbler populations (Table 1) to estimate the expected occurrence rate of each genotype within these populations, under the assumption of Hardy-Weinberg equilibrium (Table 2).

**Table 2:** Expected occurrence rate of each genotype in southern and northern populations calculated from the subspecies-specific allele frequencies (Table 1) under the assumption of Hardy-Weinberg Equilibrium and similar population size between southern and northern population

Genotype	Expected occurrence rate		
	Southern population	Northern population	Log likelihood ratio^a^
SS	91.288%	0.002%	4.659
SH	5.647%	0.038%	2.172
HS	2.780%	0.065%	1.631
SN	0.087%	0.210%	0.382
HH	0.172%	1.446%	0.924
NS	0.021%	0.622%	1.471
HN	0.003%	7.988%	3.425
NH	0.001%	13.738%	4.137
NN	0.000%	75.890%	4.926

^a^Calculated as the 10-log difference in occurrence rate (highest – lowest)

The chance of finding the genotypes SS and SH are much higher (45,000 and 148 times more likely) in a southern than in a northern population (Table 2). Likewise, each of the genotypes NN, NH and HN are > 2600 times more likely to be found in an *acredula* than in a *trochilus* population. Importantly, the occurrence probabilities of heterozygotes (HH) or mismatching homozygotes (SN, NS) were low (< 1.5%) in both the southern and northern populations (Table 2). Based on these calculations, we conclude that genotypes HH, SN and NS are extremely unlikely to originate from populations of pure *trochilus* or *acredula*.

For the following analyses we therefore assume that genotypes SS, HS and SH mostly originate from populations of allopatric *trochilus* and individuals with the genotypes NN, NH and HN from populations of allopatric *acredula*. We further assume that the genotypes HH consist of F1 hybrid offspring from ( *trochilus* x *acredula*) and originate from any of the two migratory divides, as do the genotypes SN and NS which are likely to be F2 offspring from HH x HH parents. To confirm genotypes of the supposed hybrid individuals, we genotyped these samples for the additional SNPs (23 and 412). Willow warblers that showed the same hybrid genotype for the two sets of SNPs were classified as hybrids. Three birds had mismatching genotypes for the two SNPs on the same chromosome and were classified as “undefined”. Note that this is not a technical genotyping problem, but a consequence of the nucleotides not being fixed for the alternative variants in the reference populations (Table 1).

We used a Binomial Probability Test to estimate whether the F1 heterozygotes (HH) were over-represented in migratory sampling sites.

## Results

### Genotypes in winter samples from Africa

**Fig. 2:**
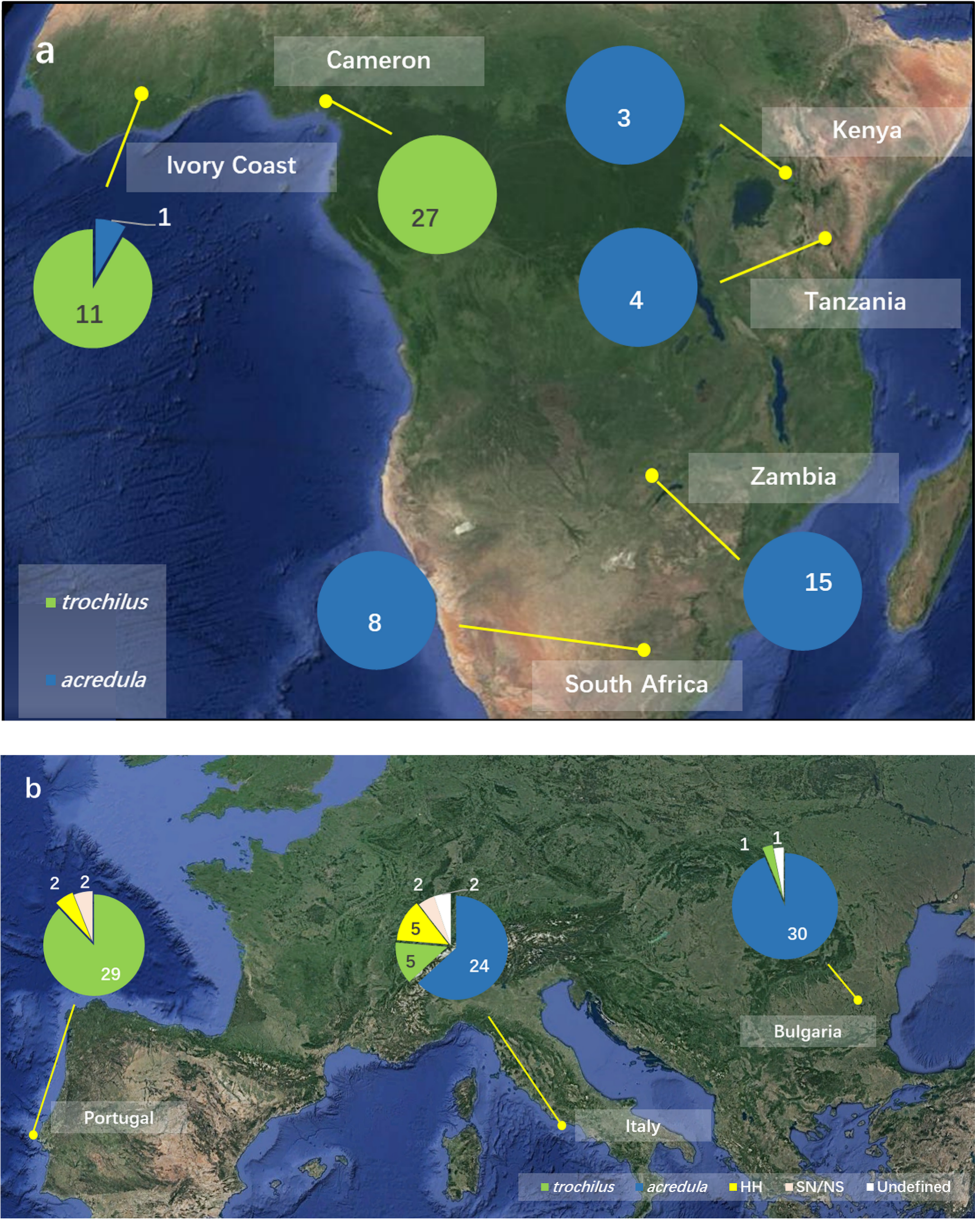
Genetic assignments of willow warblers at sampling sites in a. Africa (November – April) and b. juveniles during autumn migration (August – October) in southern Europe. Genotypes “SS”, “HS”, “SH” were assigned as the subspecies *trochilus* and “NN”, “NH”, “HN” as the subspecies *acredula*. “HH” represents F1 hybrids and “SN/NS” F2 hybrids as verified with the other two SNPs. Three potential hybrid individuals carried conflicting genotypes for SNPs within chromosomes and are shown as “Undefined”. Sample sizes: Ivory Coast, *n* = 12; Cameron, *n* = 27; Kenya, *n* = 3; Tanzania, *n* = 4; Zambia, *n* = 15; South Africa, *n* = 8; Portugal, *n* = 33; Italy, *n* = 38; Bulgaria, *n* = 32

The genotype compositions differed markedly between western compared to eastern and southern Africa (Fig. 2a). In western Africa (Ivory Coast (*n* = 12) and Cameroon (*n* = 27), 97.3% of the individuals were assigned to *trochilus*, and one individual was assigned to *acredula*. In eastern Africa (Kenya (*n* = 3), Tanzania (*n* = 4), Zambia (*n* = 15) and South Africa (*n* = 8)), all individuals were assigned to *acredula*. Among the winter samples, we did not find any individual with a presumed origin from the hybrid zones (HH, SN or NS individuals).

### Genotyping results of juvenile birds captured during autumn migration in Europe

Juveniles sampled in Portugal, Italy and Bulgaria clearly had different genotype compositions (Fig. 2b). In Portugal, 87.9% (*n* = 33) of the individuals were assigned to *trochilus*, and of the remaining, two were HH, one SN and one NS. The analyses of SNP 23 and 412 confirmed these hybrid genotypes. In Bulgaria, 93.8% (*n* = 32) of the individuals were assigned to *acredula*. Of the remaining two birds, one was assigned to *trochilus* and one as undefined genotype (northern or F2). In Italy, 63.2% (*n* = 38) of the individuals were assigned to *acredula*, 13.2% to *trochilus*, five birds were HH, one SN and one NS. The analyses of SNP 23 and 412 confirmed these hybrid genotypes. However, the genotypes of two birds could not be resolved (one was F2 or hybrid, the other southern or hybrid). By assuming a background level of heterozygote individuals (HH) of 1.5% in allopatric *trochilus* and *acredula* populations (Table 2), we observed that HH individuals were significantly over-represented in Italy (Binomial Probability Test, *p*-value = 0.00023). Based on the same assumption, the HH individuals were not significantly over-represented in Portugal (Binomial Probability Test, *p*-value = 0.07) or in Bulgaria (Binomial Probability Test, *p*-value = 0.62).

## Discussion

In this study, we used genotype assignments to investigate the autumn migratory direction of juvenile willow warblers. This indirect, though unbiased approach, allowed us to test whether juvenile F1 hybrids between the subspecies *trochilus* and *acredula* migrate along directions similar to either of the parental forms, or if they follow an intermediate route as has been proposed for hybrid willow warblers [24], and several other species having narrow migratory divides [13, 30]. We first demonstrated that the two SNP markers used for this study are highly fixed between the subspecies *trochilus* and *acredula*, and that hybrid individuals have a high likelihood of origin from the migratory divides. We tested the SNP assay on willow warbler samples from winter quarters in Africa, and found that *trochilus* and *acredula* exclusively (except for one *acredula* in Ivory Coast in April) occurred in western and eastern Africa, respectively, in line with the well-documented wintering quarters of the subspecies [11, 19, 23]. We can think of two explanations to why we did not find any hybrids among the samples from Africa. First, if hybrids tend, as predicted, to winter in Central Africa, this region is not represented among our available samples. Second, if they winter throughout the range of both subspecies, their frequency at any location would be very low and thus likely to be missed due to our small sample size (*n* = 69).

During autumn migration in southern Europe, juvenile *trochilus* and *acredula* dominated in western (Portugal) and eastern Europe (Bulgaria), respectively, also in line with the well documented autumn migration directions of these subspecies [19, 22].

Among the autumn samples, we identified seven birds as F1 hybrids. Although the two investigated SNPs are not completely fixed for the alternative alleles in allopatric *trochilus* and *acredula* populations, our calculations show that F1 individuals are expected to occur at very low frequencies in the allopatric populations (0.172% in *trochilus* and 1.446% in *acredula*). Because our references samples for allopatric *trochilus* and *acredula* were collected relatively close to the migratory divides compared to the huge allopatric ranges of the subspecies, a background frequency of hybrid genotypes < 1.5% is deemed a conservative estimate. In line with this, Lundberg et al. (2017) reported that populations most distant from the migratory divides (Denmark and UK representing the *trochilus* haplotype and southern Finland and Siberia representing the *acredula* haplotype) were completely fixed for the subspecies specific haplotypes, both for chromosome 1 and 5 [28].

In the Scandinavian migratory divide, 27% of the territorial (adult) males are hybrids (HH) or conflicting homozygotes (NS and SN) [28]. Hence, most (if not all) F1 hybrids that we recorded are likely to have originated from the migratory divides in Scandinavia or Poland. During autumn migration in southern Europe, the juvenile F1 hybrid individuals were significantly over-represented in the sample from Italy, rarely shown in Portugal and absent in Bulgaria. This result is in line with the expected intermediate route, if these hybrids originated from any of the two contact zones in Scandinavia or Poland.

The overrepresentation of heterozygous juvenile willow warblers (F1 hybrids) in Italy during autumn migration suggests that hybrids migrate along an intermediate route during their first migration relative to their parental populations, in line with the hypothesis proposed by Helbig [8]. This interpretation implies that the genes encoding migration direction, in the willow warbler presumed to be located on the blocks on chromosome 1 and 5 [28], have a co-dominant effect on the phenotype. However, in conflict to this conclusion are our records of two hybrids in Portugal, clearly resembling the routes of pure *trochilus*. Similar results were shown in the isotope studies in the flycatchers [9] and *Acrocephalus* warblers [10], where the hybrids migrated southwest as one of the parents. The two Portuguese willow warbler hybrids were captured in two different years (2011 and 2012), and thus it is unlikely that their presence in Portugal was due to unusual weather conditions. To identify which of the genes in the differentiated chromosome regions that may determine migratory direction, will require data on the migratory routes for a larger sample of hybrids, F2’s and back-crossed individuals, as well as samples from a wider geographical range. Eventually, such data may enable us to investigate the dominance pattern of the genes, as well as whether there are other overlooked genomic regions responsible for the migration direction.

The intermediate route of hybrids has been suggested to function as a post-zygotic reproductive isolation mechanism, maintaining migratory divides. In willow warblers, the intermediate route passes over the central Mediterranean Sea and the Sahara Desert, which are commonly regarded as ecological barriers [6, 7], and therefore would induce higher mortality rates than the parental routes to Africa, east or west of these barriers. However, the route across the central Mediterranean Sea is nonetheless a major migration route for some other species of songbirds, e.g. wood warblers [31] and sedge warblers [32]. It is possible that these species are better-adapted to this route than hybrid willow warblers, by for example putting on more fat reserves before crossing the Sahara. Because there is a substantial proportion of breeding willow warblers of hybrid origin in the migratory divides [28], i.e. birds that have completed the migration to and back from the wintering grounds, the annual mortality of hybrids cannot be very different from the mortality of the parental forms. From the equation in Barton and Hewitt (1985) [33], and using estimates of dispersal distances (σ ≅ 80 km) and width of the hybrid zone (250 km) [22], we can calculate that the selection against hybrids is ~ 10% per generation. This rough estimate would scale to a reduction of hybrid survival from e.g. 50 to 45%. i.e. a difference large enough to keep the width of the hybrid zone. However to statistically verify such a small difference would require survival data for > 1000 individuals.

In contrast to experienced adult migrants, as solitary migrants, juveniles have to rely on their endogenous navigation system and the inherited direction or “goal” as guidance [34]. The lack of previous experience increases the risk that they become disoriented [35–37]. We therefore expect that the direction differences observed among juveniles will be larger than among adults [38–41]. Because juveniles have no prior experience of the environment along the migration route [42], these should better represent the endogenous migratory routes of the species, and prior to selection, represent a more unbiased subject for studies of the genetic basis of migration.

## Conclusion

Our study on juvenile willow warblers supports the hypothesis that hybrids tend to follow an intermediate route during autumn migration compared to the parental populations. This finding also supports the hypothesis that the divergent blocks on chromosome 1 and 5 in willow warblers [28] may contain genes encoding migration direction. In species with known population genetic structure, such as the willow warbler, the here used qPCR-based SNP genotyping makes it possible to reveal details of the first-year migration that will be difficult to obtain with other methods. We showed that precise genotyping can be applied to DNA samples extracted from feather tips and specimen tissues, material that typically contain degraded DNA (around 100 bp), in agreement with previous studies on other species [43, 44], and therefore a convenient tool for studies combining DNA samples of different origins.

## Supplementary information

**Additional file 1: Table S1.** The DNA sample collection that have been used for SNP genotyping in this study, including the sample sizes, sample sources, sampling year and origin. **Figure S1.***F*_ST_-array results illustrating the genetic differentiation of ~ 4000 SNPs throughout the genome of willow warblers with the positions of the qPCR primers indicated. **Table S2.** The sequences of primers and probes for the four selected SNPs on chromosome 1 and 5. **Figure S2.** Examples of sample dual scatterplot of SNP genotyping results. **Figure S3.** Allele frequencies of the four selected SNPs on chromosome 1 and 5 in northern (upper pie charts) and southern populations (lower pie charts) in Scandinavia. **Table S3.** Comparison between SNP genotype results from this study and the whole-block genotype results from Lundberg et al. **Table S4.** SNP genotyping results from sites in Scandinavia. Sites with latitude < 60° are regarded as southern population. Sites with latitude > 65° are regarded as northern population. **Table S5.** SNP genotyping results from southern Europe in autumn (August–October). **Table S6.** SNP genotyping results from Africa in winter.

## Data Availability

The SNP genotyping data set are attached in the supplementary materials.
