# Supplementary material for: Autumn migration direction of juvenile willow warblers (Phylloscopus t. trochilus and P. t. acredula) and their hybrids assessed by qPCR SNP genotyping
Source: Mov Ecol. 2020 May 29;8:22. doi: 10.1186/s40462-020-00209-7 (PMC7257155; doi:10.1186/s40462-020-00209-7)
Supplement: Supplementary file 1 — Additional file 1: Table S1. The DNA sample collection that have been used for SNP genotyping in this study, including the sample sizes, sample sources, sampling year and origin. Figure S1.FST-array results illustrating the genetic differentiation of ~ 4000 SNPs throughout the genome of willow warblers with the positions of the qPCR primers indicated. Table S2. The sequences of primers and probes for the four selected SNPs on chromosome 1 and 5. Figure S2. Examples of sample dual scatterplot of SNP genotyping results. Figure S3. Allele frequencies of the four selected SNPs on chromosome 1 and 5 in northern (upper pie charts) and southern populations (lower pie charts) in Scandinavia. Table S3. Comparison between SNP genotype results from this study and the whole-block genotype results from Lundberg et al. Table S4. SNP genotyping results from sites in Scandinavia. Sites with latitude < 60° are regarded as southern population. Sites with latitude > 65° are regarded as northern population. Table S5. SNP genotyping results from southern Europe in autumn (August–October). Table S6. SNP genotyping results from Africa in winter. [file 40462_2020_209_MOESM1_ESM.docx]

**Supplementary materials**

**Table S1** The DNA sample collection that have been used for SNP genotyping in this study, including the sample sizes, sample sources, sampling year and origin.

| **Country** | **Sample size** | **Sample sources** | **Sampling year** |
| --- | --- | --- | --- |
| Sweden | 85 | The southern and northern populations are from blood, of which 21 individuals also have feather samples | 1997-2010 |
| Norway | 13 | Extracted DNA from Blood | 2006 |
| Denmark | 23 | Extracted DNA from Blood | 2005 |
| Finland | 13 | Extracted DNA from Blood | 2003 |
| Portugal | 40 | Feather | 2009-2010 |
| Italy | 44 | 25 from feather sample, 19 from blood | 2012-2013 |
| Bulgaria | 40 | Extracted DNA from Blood | 2010 |
| Zambia | 15 | Blood | 2012 |
| Cameron | 27 | Extracted DNA from Blood | 2014 |
| Tanzania | 4 | Feather | 2002 |
| South Africa | 8 | Feather | 2002 |
| Ivory Coast | 12 | Feather | 1999 |
| Kenya | 3 | Feather | 2000 |


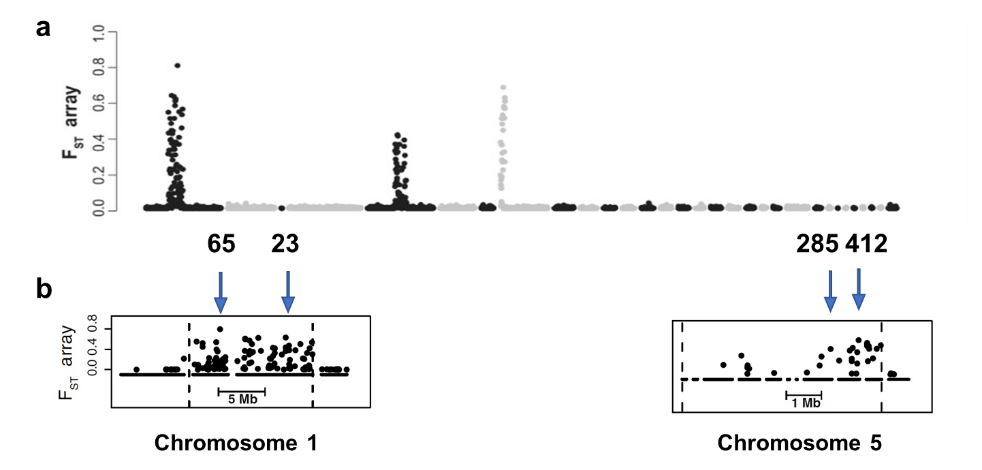


**Fig. S1** *F*_ST_-array results illustrating the genetic differentiation of ~4000 SNPs throughout the genome of willow warblers with the positions of the qPCR primers indicated.

**Table S2.** The sequences of primers and probes for the four selected SNPs on chromosome 1

and 5.

| Chromosome | SNP names |  | Base sequences | Region length |
| --- | --- | --- | --- | --- |
| Chr_1 | 23 | Forward primer | TGAAACAGAATTTTGCAGTACTTCTACCT | 112 |
|  |  | Reverse primer | GTGCTTTCTAGTATATGGAAAAAAGAGGGA |  |
|  |  | VIC probe (North) | ATTGAAATCAA**G**AATTCAG |  |
|  |  | FAM probe (South) | ATTGAAATCAA**T**AATTCAG |  |
|  | 65 | Forward primer | ACAACAACATGTAAAGCAGGAACCT | 96 |
|  |  | Reverse primer | ACCTGTTGTTAAAGGTTATGTCAATTCCT |  |
|  |  | VIC probe (North) | ACTAAATAACACTAA**A**ATTTT |  |
|  |  | FAM probe (South) | ACTAAATAACACTAA**C**ATTTT |  |
| Chr_5 | 412 | Forward primer | CATCTGGGCTCCACTTGCT | 144 |
|  |  | Reverse primer | TGTTGCCTAAATCCTGGGTTTCC |  |
|  |  | VIC probe (North) | TGCCC**G**GATTTTT |  |
|  |  | FAM probe (South) | TGCCC**A**GATTTTT |  |
|  | 285 | Forward primer | CACATTTATTAGTATTATGCCCCCAGGTA | 95 |
|  |  | Reverse primer | TATTGATTTAACAACACAGAATGCATGTGA |  |
|  |  | VIC probe (North) | CTGCATATAATCTT**A**CCTTTC |  |
|  |  | FAM probe (South) | TGCATATAATCTT**G**CCTTTC |  |


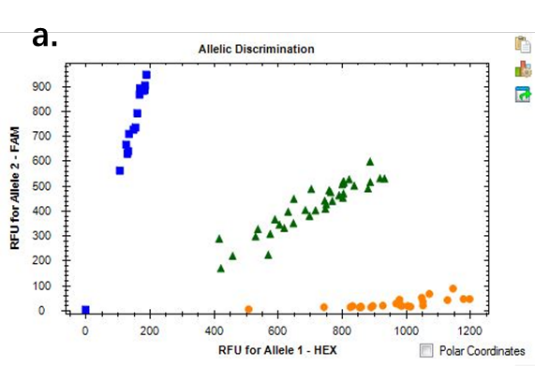

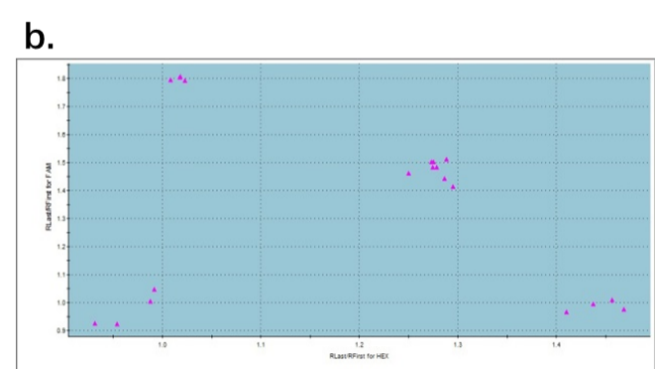


**Fig. S2** Examples of sample dual scatterplot of SNP genotyping results.


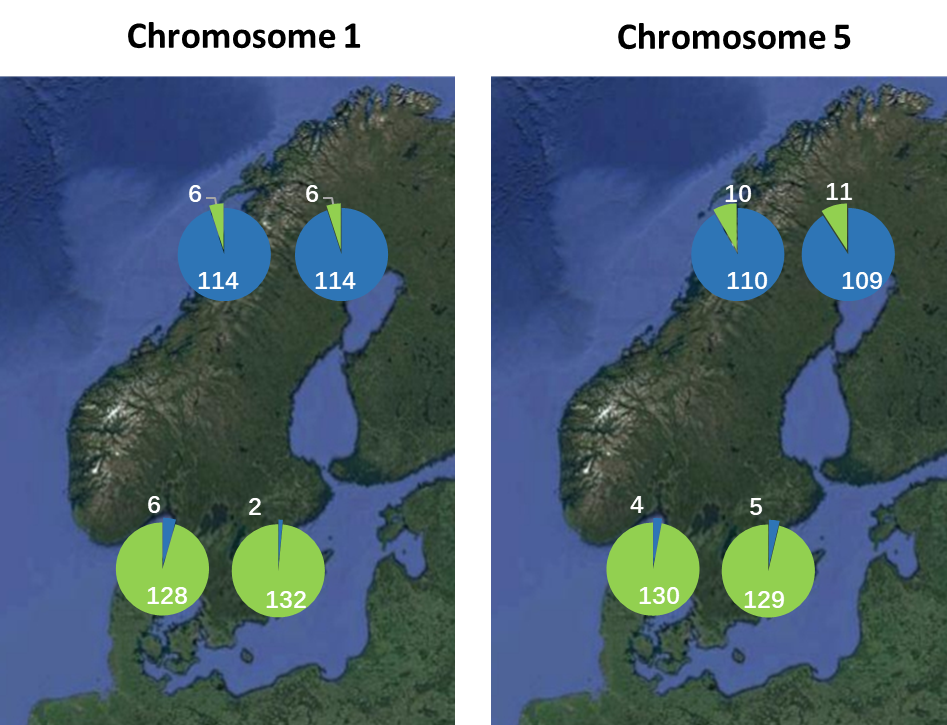


**Fig. S3** Allele frequencies of the four selected SNPs on chromosome 1 and 5 in northern (upper pie charts) and southern populations (lower pie charts) in Scandinavia.

**Table S3** Comparison between SNP genotype results from this study and the whole-block genotype results from Lundberg et al.

| SNP | chr_1 | | chr_5 | |
| --- | --- | --- | --- | --- |
|  | 23 | 65 | 285 | 412 |
| counts | 93/97 | 96/97 | 95/97 | 95/97 |
| consistency ratio | 95.9% | 99.0% | 97.9% | 97.9% |

**Table S4** SNP genotyping results from sites in Scandinavia. Sites with latitude < 60° are regarded as southern population. Sites with latitude > 65° are regarded as northern population.

| Sample name | Site name | Latitude | Longitude | SNP genotype | | | |
| --- | --- | --- | --- | --- | --- | --- | --- |
|  |  |  |  | 23 | 65 | 285 | 412 |
| 01Q/01 | Gräsmarö | 58.28 | 16.98 | H | H | S | S |
| 05B/08 | Kasted_Mose | 56.17 | 10.05 | H | S | S | S |
| 05B/12 | Kasted_Mose | 56.17 | 10.05 | H | S | S | S |
| 01A/13 | Stensoffa | 55.70 | 13.46 | H | S | S | S |
| 97E/02 | Tåkern | 58.32 | 14.82 | S | S | H | H |
| 03K/06 | Gräsmarö | 58.28 | 16.98 | S | S | H | H |
| 08A/18 | Stensoffa | 55.70 | 13.46 | S | S | H | S |
| 05C/02 | Gadevang_Mose | 55.83 | 12.47 | S | S | S | H |
| 08B/06 | Stensoffa | 55.70 | 13.46 | S | S | S | H |
| 03K/01 | Gräsmarö | 58.28 | 16.98 | S | S | S | S |
| 97A/09 | Haganäs | 56.25 | 14.67 | S | S | S | S |
| 08A/04 | Stensoffa | 55.70 | 13.46 | S | S | S | S |
| 03A/08 | Stensoffa | 55.70 | 13.46 | S | S | S | S |
| 05B/06 | Kasted_Mose | 56.17 | 10.05 | S | S | S | S |
| 05C/01 | Gadevang_Mose | 55.83 | 12.47 | S | S | S | S |
| 05C/08 | Gadevang_Mose | 55.83 | 12.47 | S | S | S | S |
| 03A/06 | Stensoffa | 55.70 | 13.46 | S | S | S | S |
| 01A/12 | Stensoffa | 55.70 | 13.46 | S | S | S | S |
| 96A/05 | Stensoffa | 55.70 | 13.46 | S | S | S | S |
| 97E/04 | Tåkern | 58.32 | 14.82 | S | S | S | S |
| 97A/08 | Haganäs | 56.25 | 14.67 | S | S | S | S |
| 01A/02 | Stensoffa | 55.70 | 13.46 | S | S | S | S |
| 02A/08 | Stensoffa | 55.70 | 13.46 | S | S | S | S |
| 03K/02 | Gräsmarö | 58.28 | 16.98 | S | S | S | S |
| 08B/05 | Stensoffa | 55.70 | 13.46 | S | S | S | S |
| 08XA/07 | Stensoffa | 55.70 | 13.46 | S | S | S | S |
| 10A/07 | Stensoffa | 55.70 | 13.46 | S | S | S | S |
| 97A/11 | Haganäs | 56.25 | 14.67 | S | S | S | S |
| 97E/05 | Tåkern | 58.32 | 14.82 | S | S | S | S |
| 97E/10 | Tåkern | 58.32 | 14.82 | S | S | S | S |
| 97E/09 | Tåkern | 58.32 | 14.82 | S | S | S | S |
| 01Q/04 | Gräsmarö | 58.28 | 16.98 | S | S | S | S |
| 96A/15 | Stensoffa | 55.70 | 13.46 | S | S | S | S |
| 02A/02 | Stensoffa | 55.70 | 13.46 | S | S | S | S |
| 02A/09 | Stensoffa | 55.70 | 13.46 | S | S | S | S |
| 97A/12 | Haganäs | 56.25 | 14.67 | S | S | S | S |
| 05B/01 | Kasted_Mose | 56.17 | 10.05 | S | S | S | S |
| 05B/02 | Kasted_Mose | 56.17 | 10.05 | S | S | S | S |
| 05B/03 | Kasted_Mose | 56.17 | 10.05 | S | S | S | S |
| 05B/04 | Kasted_Mose | 56.17 | 10.05 | S | S | S | S |
| 05B/05 | Kasted_Mose | 56.17 | 10.05 | S | S | S | S |
| 05B/07 | Kasted_Mose | 56.17 | 10.05 | S | S | S | S |
| 05B/09 | Kasted_Mose | 56.17 | 10.05 | S | S | S | S |
| 05B/10 | Kasted_Mose | 56.17 | 10.05 | S | S | S | S |
| 05B/11 | Kasted_Mose | 56.17 | 10.05 | S | S | S | S |
| 05C/03 | Gadevang_Mose | 55.83 | 12.47 | S | S | S | S |
| 05C/04 | Gadevang_Mose | 55.83 | 12.47 | S | S | S | S |
| 05C/05 | Gadevang_Mose | 55.83 | 12.47 | S | S | S | S |
| 05C/06 | Gadevang_Mose | 55.83 | 12.47 | S | S | S | S |
| 05C/07 | Gadevang_Mose | 55.83 | 12.47 | S | S | S | S |
| 05C/09 | Gadevang_Mose | 55.83 | 12.47 | S | S | S | S |
| 05C/10 | Gadevang_Mose | 55.83 | 12.47 | S | S | S | S |
| 05C/11 | Gadevang_Mose | 55.83 | 12.47 | S | S | S | S |
| 08A/06 | Stensoffa | 55.70 | 13.46 | S | S | S | S |
| 08B/08 | Stensoffa | 55.70 | 13.46 | S | S | S | S |
| 02A/03 | Stensoffa | 55.70 | 13.46 | S | S | S | S |
| 02A/11 | Stensoffa | 55.70 | 13.46 | S | S | S | S |
| 02A/13 | Stensoffa | 55.70 | 13.46 | S | S | S | S |
| 08A/05 | Stensoffa | 55.70 | 13.46 | S | S | S | S |
| 08A/13 | Stensoffa | 55.70 | 13.46 | S | S | S | S |
| 96B/08 | Kvismaren | 59.17 | 15.42 | S | S | S | S |
| 96B/05 | Kvismaren | 59.17 | 15.42 | S | S | S | S |
| 03J/06 | Smedstorp | 59.58 | 14.98 | S | S | S | S |
| 03J/07 | Smedstorp | 59.58 | 14.98 | H | S | H | H |
| 02F/09 | Smedstorp | 59.58 | 14.98 | S | H | S | S |
| 03J/02 | Smedstorp | 59.58 | 14.98 | H | S | S | S |
| 01P/02 | Stensoffa | 55.70 | 13.46 | S | S | S | S |
| 06D/06 | Kjöllefjord | 70.94 | 27.42 | H | H | H | H |
| 01J/09 | Krokvik | 67.95 | 20.02 | H | H | N | N |
| 01J/05 | Krokvik | 67.95 | 20.02 | H | H | N | N |
| 06D/04 | Kjöllefjord | 70.94 | 27.42 | H | H | N | N |
| 06D/07 | Kjöllefjord | 70.94 | 27.42 | H | H | N | N |
| 06D/05 | Kjöllefjord | 70.94 | 27.42 | H | H | N | N |
| 96M/05 | Ammarnäs | 65.95 | 16.12 | N | N | H | H |
| 01O/03 | Kallax | 65.53 | 22.13 | N | N | H | H |
| 03I/03 | Lurio | 67.23 | 27.55 | N | N | H | H |
| 06E/08 | Tana_Bru | 70.18 | 28.20 | N | N | H | H |
| 01K/01 | Tornehamn | 68.43 | 18.58 | N | N | H | H |
| 01M/03 | Kaisepakte | 68.28 | 19.32 | N | N | H | H |
| 01N/07 | Altajärvi | 67.83 | 20.53 | N | N | H | H |
| 03I/02 | Lurio | 67.23 | 27.55 | N | N | H | H |
| 10K/07 | Ekorrsjö | 64.49 | 19.07 | N | N | H | H |
| 96M/01 | Ammarnäs | 65.95 | 16.12 | N | N | N | H |
| 97N/14 | Gällivare | 67.22 | 20.80 | N | N | N | N |
| 01N/05 | Altajärvi | 67.83 | 20.53 | N | N | N | N |
| 03H/01 | Ruka | 66.18 | 29.42 | N | N | N | N |
| 03H/06 | Ruka | 66.18 | 29.42 | N | N | N | N |
| 03I/01 | Lurio | 67.23 | 27.55 | N | N | N | N |
| 03I/10 | Lurio | 67.23 | 27.55 | N | N | N | N |
| 06D/03 | Kjöllefjord | 70.94 | 27.42 | N | N | N | N |
| 01K/05 | Tornehamn | 68.43 | 18.58 | N | N | N | N |
| 03H/04 | Ruka | 66.18 | 29.42 | N | N | N | N |
| 03H/08 | Ruka | 66.18 | 29.42 | N | N | N | N |
| 97L/06 | Piteå | 65.03 | 21.45 | N | N | N | N |
| 03I/04 | Lurio | 67.23 | 27.55 | N | N | N | N |
| 01M/08 | Kaisepakte | 68.28 | 19.32 | N | N | N | N |
| 01L/19 | Stordalen | 68.33 | 19.10 | N | N | N | N |
| 01L/20 | Stordalen | 68.33 | 19.10 | N | N | N | N |
| 97N/04 | Gällivare | 67.22 | 20.80 | N | N | N | N |
| 01J/07 | Krokvik | 67.95 | 20.02 | N | N | N | N |
| 01K/08 | Tornehamn | 68.43 | 18.58 | N | N | N | N |
| 01N/08 | Altajärvi | 67.83 | 20.53 | N | N | N | N |
| 97M/03 | Kukkola | 65.96 | 24.03 | N | N | N | N |
| 97N/03 | Gällivare | 67.22 | 20.80 | N | N | N | N |
| 01L/14 | Stordalen | 68.33 | 19.10 | N | N | N | N |
| 01J/04 | Krokvik | 67.95 | 20.02 | N | N | N | N |
| 01K/10 | Tornehamn | 68.43 | 18.58 | N | N | N | N |
| 01O/01 | Kallax | 65.53 | 22.13 | N | N | N | N |
| 03H/09 | Ruka | 66.18 | 29.42 | N | N | N | N |
| 03I/06 | Lurio | 67.23 | 27.55 | N | N | N | N |
| 03I/07 | Lurio | 67.23 | 27.55 | N | N | N | N |
| 06D/02 | Kjöllefjord | 70.94 | 27.42 | N | N | N | N |
| 06D/10 | Kjöllefjord | 70.94 | 27.42 | N | N | N | N |
| 06E/01 | Tana_Bru | 70.18 | 28.20 | N | N | N | N |
| 06E/02 | Tana_Bru | 70.18 | 28.20 | N | N | N | N |
| 06E/04 | Tana_Bru | 70.18 | 28.20 | N | N | N | N |
| 06E/05 | Tana_Bru | 70.18 | 28.20 | N | N | N | N |
| 06E/07 | Tana_Bru | 70.18 | 28.20 | N | N | N | N |
| 97M/01 | Kukkola | 65.96 | 24.03 | N | N | N | N |
| 97L/03 | Piteå | 65.03 | 21.45 | N | N | N | N |
| 97M/07 | Kukkola | 65.96 | 24.03 | N | N | N | N |
| 01O/08 | Kallax | 65.53 | 22.13 | N | N | N | N |
| 10J/09 | Granö | 64.27 | 19.42 | N | N | N | N |
| 10L/02 | Gunnarn | 65.00 | 17.71 | N | N | N | N |
| 01N/12 | Altajärvi | 67.83 | 20.53 | N | N | N | N |
| 01O/04 | Kallax | 65.53 | 22.13 | N | N | N | N |
| 03H/10 | Ruka | 66.18 | 29.42 | N | N | N | N |

**Table S5** SNP genotyping results from southern Europe in autumn (August–October).

| Sample name | Country | SNP genotypes | | | | Assignment |
| --- | --- | --- | --- | --- | --- | --- |
|  |  | 23 | 65 | 285 | 412 |  |
| 6G0646 | Italy |  | N | N |  | northern pop. |
| 6G0653 | Italy |  | N | N |  | northern pop. |
| 1A78028 | Italy | S | S | N | N | F2 |
| 6G0640 | Italy |  | N | H |  | northern pop. |
| 6G0622 | Italy |  | H | N |  | northern pop. |
| 6G0639 | Italy |  | H | N |  | northern pop. |
| 6G0603 | Italy |  | N | N |  | northern pop. |
| 6G0606 | Italy |  | N | N |  | northern pop. |
| 6G0607 | Italy |  | N | N |  | northern pop. |
| 6G0619 | Italy |  | N | N |  | northern pop. |
| 6G0644 | Italy |  | N | N |  | northern pop. |
| 6G0616 | Italy |  | S | H |  | southern pop. |
| 6G0636 | Italy |  | N | H |  | northern pop. |
| 3A15807 | Italy |  | S | S |  | southern pop. |
| 6G0623 | Italy |  | H | N |  | northern pop. |
| 6G0615 | Italy |  | H | N |  | northern pop. |
| 6G0656 | Italy |  | H | N |  | northern pop. |
| 6G0612 | Italy |  | N | N |  | northern pop. |
| 6G0618 | Italy |  | N | N |  | northern pop. |
| 6G0654 | Italy |  | N | N |  | northern pop. |
| 6G0655 | Italy | S | S | N | N | F2 |
| 1A78029 | Italy |  | S | H |  | southern pop. |
| 6G0601 | Italy | H | H | H | H | heterozygote |
| 6G0649 | Italy | H | H | H | H | heterozygote |
| 6G0611 | Italy | H | H | H | S | Undefined |
| 659 | Italy |  | N | N |  | northern pop. |
| 621 | Italy |  | H | N |  | northern pop. |
| 508 | Italy | S | S | N | H | Undefined |
| 515 | Italy | H | H | H | H | heterozygote |
| 658 | Italy | H | H | H | H | heterozygote |
| 517 | Italy | H | H | H | H | heterozygote |
| 625 | Italy |  | N | N |  | northern pop. |
| 637 | Italy |  | N | N |  | northern pop. |
| 657 | Italy |  | N | N |  | northern pop. |
| 509 | Italy |  | S | S |  | southern pop. |
| 502 | Italy |  | N | N |  | northern pop. |
| 510 | Italy |  | N | N |  | northern pop. |
| 511 | Italy |  | N | N |  | northern pop. |
| 512 | Italy |  | N | N |  | northern pop. |
| 634 | Italy |  | N | N |  | northern pop. |
| 660 | Italy |  | N | N |  | northern pop. |
| 503 | Italy |  | S | H |  | southern pop. |
| 506 | Italy | H | H | H | H | heterozygote |
| 504 | Italy |  | S | H |  | southern pop. |
| 079807 | Portugal |  | S | S |  | southern pop. |
| 089873 | Portugal |  | S | S |  | southern pop. |
| 079874 | Portugal |  | S | S |  | southern pop. |
| 079808 | Portugal |  | S | S |  | southern pop. |
| 072387 | Portugal |  | S | S |  | southern pop. |
| 072389 | Portugal |  | S | S |  | southern pop. |
| 079810 | Portugal |  | S | S |  | southern pop. |
| 079873 | Portugal |  | S | S |  | southern pop. |
| 079809 | Portugal |  | S | S |  | southern pop. |
| 072392 | Portugal |  | S | S |  | southern pop. |
| 079871 | Portugal |  | S | S |  | southern pop. |
| 089868 | Portugal |  | S | S |  | southern pop. |
| 089869 | Portugal |  | S | S |  | southern pop. |
| 079875 | Portugal | H | H | H | H | heterozygote |
| 079872 | Portugal |  | S | S |  | southern pop. |
| 079811 | Portugal |  | S | H |  | southern pop. |
| 089875 | Portugal | H | H | H | H | heterozygote |
| 089874 | Portugal |  | S | S |  | southern pop. |
| 072390 | Portugal |  | S | S |  | southern pop. |
| 072388 | Portugal |  | S | S |  | southern pop. |
| 079765 | Portugal |  | H | S |  | southern pop. |
| 079766 | Portugal |  | S | S |  | southern pop. |
| 079767 | Portugal |  | S | S |  | southern pop. |
| 079768 | Portugal |  | S | S |  | southern pop. |
| 079769 | Portugal |  | S | S |  | southern pop. |
| 079912 | Portugal |  | S | S |  | southern pop. |
| 079913 | Portugal |  | S | S |  | southern pop. |
| 079914 | Portugal |  | S | S |  | southern pop. |
| 079915 | Portugal |  | S | S |  | southern pop. |
| 079916 | Portugal |  | S | S |  | southern pop. |
| 089840 | Portugal |  | S | S |  | southern pop. |
| 089841 | Portugal |  | S | S |  | southern pop. |
| 089842 | Portugal |  | S | S |  | southern pop. |
| 089843 | Portugal |  | S | S |  | southern pop. |
| 089844 | Portugal |  | S | H |  | southern pop. |
| 089846 | Portugal |  | S | S |  | southern pop. |
| 089847 | Portugal |  | S | S |  | southern pop. |
| 089848 | Portugal |  | S | S |  | southern pop. |
| 089849 | Portugal | S | S | N | N | F2 |
| 089850 | Portugal | S | S | N | N | F2 |
| 44 | Bulgaria |  | N | H |  | northern pop. |
| 45 | Bulgaria |  | N | N |  | northern pop. |
| 48 | Bulgaria |  | N | N |  | northern pop. |
| 54 | Bulgaria |  | N | N |  | northern pop. |
| 58 | Bulgaria |  | N | N |  | northern pop. |
| 62 | Bulgaria |  | N | N |  | northern pop. |
| 80 | Bulgaria |  | N | N |  | northern pop. |
| 91 | Bulgaria |  | N | N |  | northern pop. |
| 94 | Bulgaria |  | N | N |  | northern pop. |
| 98 | Bulgaria |  | N | H |  | northern pop. |
| 101 | Bulgaria |  | N | N |  | northern pop. |
| 107 | Bulgaria |  | N | N |  | northern pop. |
| 145 | Bulgaria |  | N | N |  | northern pop. |
| 146 | Bulgaria |  | N | N |  | northern pop. |
| 148 | Bulgaria |  | N | N |  | northern pop. |
| 150 | Bulgaria |  | S | H |  | southern pop. |
| 151 | Bulgaria |  | N | N |  | northern pop. |
| 165 | Bulgaria |  | N | N |  | northern pop. |
| 173 | Bulgaria |  | N | N |  | northern pop. |
| 188 | Bulgaria |  | N | N |  | northern pop. |
| 209 | Bulgaria |  | N | N |  | northern pop. |
| 212 | Bulgaria |  | N | N |  | northern pop. |
| 213 | Bulgaria |  | N | N |  | northern pop. |
| 214 | Bulgaria |  | N | N |  | northern pop. |
| 219 | Bulgaria |  | N | N |  | northern pop. |
| 222 | Bulgaria |  | N | N |  | northern pop. |
| 226 | Bulgaria |  | N | N |  | northern pop. |
| 234 | Bulgaria |  | N | N |  | northern pop. |
| 243 | Bulgaria |  | N | N |  | northern pop. |
| 244 | Bulgaria |  | N | N |  | northern pop. |
| 248 | Bulgaria |  | N | N |  | northern pop. |
| 251 | Bulgaria |  | N | N |  | northern pop. |
| 254 | Bulgaria |  | N | N |  | northern pop. |
| 257 | Bulgaria |  | N | N |  | northern pop. |
| 262 | Bulgaria |  | N | N |  | northern pop. |
| 264 | Bulgaria |  | N | N |  | northern pop. |
| 271 | Bulgaria |  | N | N |  | northern pop. |
| 268 | Bulgaria |  | N | N |  | northern pop. |
| 275 | Bulgaria | N | N | S | H | Undefined |
| 278 | Bulgaria |  | N | H |  | northern pop. |

**Table S6** SNP genotyping results from Africa in winter.

| Sample name | Country | SNP genotypes | | Assignment |
| --- | --- | --- | --- | --- |
|  |  | 65 | 285 |  |
| Z69 | Zambia | N | N | northern pop. |
| Z74 | Zambia | N | H | northern pop. |
| Z60 | Zambia | N | N | northern pop. |
| Z65 | Zambia | N | N | northern pop. |
| Z72 | Zambia | N | N | northern pop. |
| Z73 | Zambia | N | N | northern pop. |
| Z61 | Zambia | N | N | northern pop. |
| Z62 | Zambia | N | N | northern pop. |
| Z63 | Zambia | N | N | northern pop. |
| Z64 | Zambia | N | N | northern pop. |
| Z67 | Zambia | N | N | northern pop. |
| Z68 | Zambia | N | N | northern pop. |
| Z71 | Zambia | H | N | northern pop. |
| Z75 | Zambia | N | N | northern pop. |
| Z76 | Zambia | N | N | northern pop. |
| K39204 | Kenya | N | N | northern pop. |
| K39205 | Kenya | N | N | northern pop. |
| 8N 55600 | Kenya | N | N | northern pop. |
| 446 | Tanzania | N | N | northern pop. |
| T33467 | Tanzania | N | N | northern pop. |
| T33468 | Tanzania | H | N | northern pop. |
| 448 | Tanzania | N | N | northern pop. |
| AE28933 | South Africa | N | N | northern pop. |
| AE28932 | South Africa | N | H | northern pop. |
| AE28954 | South Africa | N | N | northern pop. |
| AF60012 | South Africa | N | N | northern pop. |
| W17819 | South Africa | N | N | northern pop. |
| W17824 | South Africa | N | H | northern pop. |
| W17825 | South Africa | N | N | northern pop. |
| W17826 | South Africa | N | N | northern pop. |
| BE7350 | Ivory Coast | S | S | southern pop. |
| BE7351 | Ivory Coast | S | S | southern pop. |
| BE7352 | Ivory Coast | S | S | southern pop. |
| BE7353 | Ivory Coast | N | N | southern pop. |
| BE7354 | Ivory Coast | S | S | southern pop. |
| BE7355 | Ivory Coast | S | S | southern pop. |
| BE7356 | Ivory Coast | S | S | southern pop. |
| BE7357 | Ivory Coast | S | H | southern pop. |
| BE7358 | Ivory Coast | S | S | southern pop. |
| BE7359 | Ivory Coast | S | S | southern pop. |
| BE7360 | Ivory Coast | S | S | southern pop. |
| BE7361 | Ivory Coast | S | H | southern pop. |
| 405 | Cameron | S | S | southern pop. |
| 411 | Cameron | S | S | southern pop. |
| 412 | Cameron | H | S | southern pop. |
| 420 | Cameron | S | S | southern pop. |
| 425 | Cameron | S | S | southern pop. |
| 426 | Cameron | S | H | southern pop. |
| 427 | Cameron | H | S | southern pop. |
| 429 | Cameron | H | S | southern pop. |
| 432 | Cameron | S | H | southern pop. |
| 438 | Cameron | S | S | southern pop. |
| 439 | Cameron | S | S | southern pop. |
| 442 | Cameron | S | H | southern pop. |
| 443 | Cameron | S | S | southern pop. |
| 444 | Cameron | S | H | southern pop. |
| 448 | Cameron | S | S | southern pop. |
| 449 | Cameron | S | S | southern pop. |
| 452 | Cameron | S | H | southern pop. |
| 456 | Cameron | S | S | southern pop. |
| 466 | Cameron | S | S | southern pop. |
| 468 | Cameron | S | H | southern pop. |
| 491 | Cameron | S | S | southern pop. |
| 494 | Cameron | H | S | southern pop. |
| 501 | Cameron | S | H | southern pop. |
| 502 | Cameron | S | S | southern pop. |
| 505 | Cameron | S | S | southern pop. |
| 509 | Cameron | S | S | southern pop. |
| 518 | Cameron | S | S | southern pop. |
